# Working-Class Support and Representation

**Published:** 1914-06-20

**Authors:** 


### WORKING-CLASS SUPPORT AND REPRESENTATION.

When the National Insurance Act was passed it was felt to be possible that groups of the working classes up and down the country, seeing that every workman under the Act would be compelled to pay a fixed weekly sum to National Insurance, might decide to discountenance weekly subscriptions in the workshops for the voluntary hospitals. We are glad to find that this feeling has not been justified by results, for the working man is shrewd enough to have grasped that without the aid of the voluntary hospitals National Insurance must soon be in default. The next important demonstration was that of the managers of the voluntary hospitals, who felt it was their clear policy, as it was also their duty, to prepare to treat all cases severe enough to require in-patient treatment, and at the same time to refer all trivial and ordinary cases amongst the out-patients to the panel practitioners for treatment.

At first this policy of refusal of treatment to trivial cases by voluntary hospitals was not apprehended or understood by the working classes. Indeed, they felt that they now had to pay a regular weekly sum for medical aid through the National Insurance Act and had been promised by Mr. Lloyd George adequate medical treatment in return, which they were not receiving. The working man's only hope of adequate medical treatment was through the voluntary hospitals. When, therefore, the working classes became awake to the fact that, like themselves, the voluntary hospitals were likely to lose rather than to gain through the institution of National Insurance, their sporting instincts were aroused and their sympathies and gratitude were given to the voluntary hospitals.

The financial results to the voluntary hospitals have proved encouraging; for whereas the work cast upon them has materially increased, the contributions of the working classes to these institutions have, as a rule, tended to increase rather than to diminish during the last two years. In some voluntary hospitals ordinary revenue during the same period has either remained stationary or diminished in amount. But the working men have not appreciably wavered anywhere; on the contrary, they have made often greater efforts, which have yielded increased funds for the voluntary hospitals. It is reasonable to hope from this evidence that the working classes will continue to support the hospitals to a greater extent year by year. This is a most encouraging fact which augurs well for the future of voluntary hospitals and the working classes, and is most creditable to the workers everywhere. In the case of a few hospitals the working classes subscribe half the revenue. There is reason to hope that these examples will be followed in many places, because the workers, both men and women, are fully aware that without the help of the hospitals they cannot maintain their earning power to the fullest advantage. We cannot follow this line of inquiry further to-day, but we have said enough to show that the question of working men's representation among the governors and upon the managing committees of the hospitals must soon become a question of real importance. In the case of some voluntary hospitals, the working-class representation is absurdly large. In others it is far too small, or does not exist at all.

This question of artisan representation in hospital management is one which demands examination and the formulation of some carefully thought out proposals, so that rules may be laid down on an equitable basis that may be agreeable to all parties. We hope that at the meeting of the British Hospitals' Association which assembles in Newcastle this week an opportunity may be found to discuss this question in all its bearings, with a view to the formulation of model rules which can be gradually brought into general use. We are engaged upon this question of artisan representation in hospitals at the moment. We should welcome any suggestions, experiences, or ideas that our readers may be moved promptly to send us.

